# Evaluation of Swine Protection with Three Commercial Foot-and-Mouth Disease Vaccines against Heterologous Challenge with Type A ASIA/G-VII Lineage Viruses

**DOI:** 10.3390/vaccines12050476

**Published:** 2024-04-29

**Authors:** Seon Woo Kim, Seung Heon Lee, Ha-Hyun Kim, Sung-Ho Shin, Sang-Hyun Park, Jong-Hyeon Park, Jaejo Kim, Choi-Kyu Park

**Affiliations:** 1 Animal and Plant Quarantine Agency, 177 Hyeoksin 8-ro, Gimcheon City 39660, Gyeongsangbuk-do, Republic of Korea; oneway0118@korea.kr (S.W.K.); seungheon0117@korea.kr (S.H.L.); imshin121@korea.kr (S.-H.S.); shpark0205@korea.kr (S.-H.P.); parkjhvet@korea.kr (J.-H.P.); 2 Laboratory Animal Facility, Chonnam National University Medical School, Hwasun-gun 58128, Jeollanam-do, Republic of Korea; hahyun@jnu.ac.kr; 3 Animal Disease Intervention Center, College of Veterinary Medicine, Kyungpook National University, 80, Daehak-ro, Buk-gu, Daegu 41566, Republic of Korea

**Keywords:** foot-and-mouth disease, vaccination, efficacy test, vaccine matching, virus neutralization antibody, vaccination challenge test

## Abstract

Outbreaks caused by foot-and-mouth disease (FMD) A/ASIA/G-VII lineage viruses have often occurred in Middle Eastern and Southeast Asian countries since 2015. Because A/ASIA/G-VII lineage viruses are reported to have distinct antigenic relatedness with available commercial FMD vaccine strains, it is necessary to investigate whether inoculation with vaccines used in Korea could confer cross-protection against A/ASIA/G-VII lineage viruses. In the present study, we conducted two vaccination challenge trials to evaluate the efficacy of three commercial FMD vaccines (O/Manisa + O/3039 + A/Iraq, O/Campos + A/Cruzeiro + A/2001, and O/Primorsky + A/Zabaikalsky) against heterologous challenge with ASIA/G-VII lineage viruses (A/TUR/13/2017 or A/BHU/3/2017 strains) in pigs. In each trial, clinical signs, viremia, and salivary shedding of virus were measured for 7 days after challenge. In summary, the O/Campos + A/Cruzeiro + A/2001 vaccine provided full protection against two A/ASIA/G-VII lineage viruses in vaccinated pigs, where significant protection was observed. Although unprotected animals were observed in groups vaccinated with O/Manisa + O/3039 + A/Iraq or O/Primorsky + A/Zabaikalsky vaccines, the clinical scores and viral RNA levels in the sera and oral swabs of vaccinated animals were significantly lower than those of unvaccinated controls.

## 1. Introduction

Foot-and-mouth disease (FMD) is an economically important contagious vesicular disease that predominantly affects cloven-hoofed animals such as cattle, pigs, sheep, and goats. FMD virus (FMDV), the causative agent of FMD, is an icosahedral single-stranded RNA virus that belongs to the genus *Aphthovirus*, family *Picornaviridae*. The virus consists of seven immunologically distinguishable serotypes of FMDV: O, A, C, Asia 1, and Southern African Territory (SAT) 1, SAT 2 and SAT 3. These serotypes include multiple variants with different antigenic and genetic features [1]. In this regard, the antigenic diversity within and between each serotype of FMDV often causes incomplete cross-protection in animals vaccinated with the same serotype of FMD vaccine [2].

Type A is considered antigenically and genetically the most diverse FMDV serotype and has three topotypes, ASIA, AFRICA, and Europe–South America (EURO-SA), with multiple diverse lineages and sublineages [3,4]. Among the three topotypes, the ASIA topotype has been reported to be prevalent in endemic countries in Asia and is responsible for sporadic incursion into North Africa [5]. Recently, the A/ASIA/G-VII lineage (A/G-VII), which originated from several countries classified into FMD pool 2 [6,7], has been noted as one of the most dominant FMDV strains in the Middle East since 2015 [5]. In view of past FMD outbreaks caused by various cross-border transmissions, the trans-pool spreading of the A/G-VII lineage is continuously of particular concern in the Middle East and beyond other FMD pools [3].

Although vaccination is one of the major measures used for FMD control to alleviate the impact of clinical disease, it is essential to select vaccines that could confer cross-protection against new strains [8]. For appropriate FMD vaccine strain selection, in vitro vaccine matching tests are often conducted to estimate the serological relatedness (r_1_-value) between field and vaccine viruses with bovine vaccinal serum [8,9]. An r_1_-value greater than 0.3 is considered a sufficient close serological relatedness between field and vaccine strains such that the vaccine containing the vaccine strain is likely to confer cross-protection against challenges with the field virus. However, protection depends on both the cross-reactivities and the potency of the vaccine [2,9,10].

Three commercial vaccines recently used for routine vaccination in Korea are inactivated bivalent (serotype O and A) FMDV vaccines. The Korean government requires the potency of these bivalent oil emulsion vaccines to meet more than six 50% protective dose (PD_50_)/dose because higher potency vaccines could induce a wider spectrum of cross-protection and earlier protection according to a study by Brehm et al. [2]. Since 2011, FMD outbreaks have occurred eight times including the last outbreak, caused by O/ME-SA/Ind-2001e lineage virus in 2023 in Korea, and immediate eradication through mass vaccination and other control measures has been repeated [11]. Because of genetic differences between past and new isolates through phylogenetic analysis, it is strongly assumed that outbreaks were caused by introduction from FMD-endemic neighboring countries around Korea [12]. Therefore, studying the suitability of vaccines is necessary to address the possible threat of the introduction of genetically distinct FMDV strains, such as A/ASIA/G-VII lineage viruses, into Korea in the future.

In this study, we performed in vitro vaccine matching tests between vaccines and A/ASIA/G-VII lineage strains using bovine vaccinated sera. In addition, vaccination challenge experiments were conducted to evaluate whether the commercial vaccines used in Korea would confer effective cross-protection to vaccinated pigs against two A/ASIA/G-VII lineage strains originating from South Asia, regardless of the results of vaccine matching.

## 2. Materials and Methods

### 2.1. Cells, Viruses, and Vaccines

Porcine kidney (LFBK) cells were used to culture FMDV in Dulbecco’s modified Eagle’s medium. LFBK cells obtained from the Plum Island Animal Disease Center (New York, NY, USA) were used for in vitro studies. For the separate challenge, A/TUR/13/2017 and A/BHU/3/2017 were selected considering genetic distance and r_1_-values among four A/ASIA/G-VII viruses isolated in 2017 provided by the Pirbright Institute (Woking, UK) [13]. Vesicular fluid from challenged pigs was used for the challenge study, after the virulence of both viruses in pigs, such as manifestation of typical FMD lesions on feet, was demonstrated through a pilot study. The vaccine strains, A_22_/IRQ/24/64 (A/Iraq), A_24_/Cruzeiro/BRA/55 (A/Cruzeiro) and A/ARG/2001 (A/2001), and A/Zabaikalsky/RUS/2013 (A/Zabaikalsky) for the virus neutralization test (VNT) were provided by Boehringer Ingelheim (BI, Saint-Priest, France), Sevicio Nacional de Sanidad Animal (SENASA, Martínez, Argentina) and the Federal Center for Animal Health (FGBI “ARRIAH”, Vladimir, Russia), respectively.

Three bivalent commercial vaccines, O/Manisa + O/3039 + A/Iraq formulated as a double oil emulsion (DOE), O/Primorsky + A/Zabaikalsky formulated as a DOE, and O/Campos + A/Cruzeiro + A/2001 formulated as a single oil emulsion, were purchased from BI, ARRIAH, and Biogenesis Bago (Buenos Aires, Argentina), respectively.

### 2.2. Vaccine Matching (Determination of r_1_-Values)

The r_1_-values between the vaccine strains and the A/ASIA/G-VII field isolates were measured by two-dimensional VNT (2D-VNT) using bovine vaccinal sera as described previously [9,14,15]. Pooled sera of five cattle vaccinated with a vaccine (A/Iraq, A/Cruzeiro, and A/Zabaikalsky) were used for vaccine matching for two A/ASIA/G-VII isolates. Vaccine matching test with A/2001 was not conducted because neither a monovalent A/2001 vaccine nor bovine vaccinal sera against A/2001 could be obtained. Briefly, to measure the levels of cross-reactivity between antisera raised against different vaccine antigens (A/Iraq, A/Cruzeiro, and A/Zabaikalsky) and A/ASIA/G-VII field isolates at 100 TCID_50_, VN titers against homologous and heterologous viruses were obtained by chequerboard titration of virus against vaccine serum along with a back-titration of virus alone. VN titers against five dilutions of virus ranging from 10 to 1000 TCID_50_ were plotted with virus titers fixed by back-titration, and the expected VN titers at 100 TCID_50_ were subsequently calculated via linear regression. The r_1_-values are derived by dividing reciprocal arithmetic titer against field isolate by reciprocal arithmetic titer against vaccine virus. To calculate the r_1_-value for each isolate, vaccine matching tests were carried out three times. If the r_1_-value is greater than 0.3, the field isolate is considered matched with the vaccine strain.

### 2.3. Animal Experiments for the Vaccine Efficacy Study

Vaccine efficacy trials were conducted at an animal biosafety level 3 facility of the Animal and Plant Quarantine Agency (APQA) following protocols approved by the Institutional Animal Care and Use Committee of the APQA (IACUC No. 2021-571). All test animals were acclimated for at least 1 week prior to the experiments.

To evaluate the protective efficacy of the FMD vaccine against A/ASIA/G-VII field isolates, in vivo vaccination challenge experiments were performed in pigs. In each trial, fifteen FMD-free crossbred pigs (eight weeks of age) were divided into four groups, consisting of three vaccinated groups (n = 4) and one unvaccinated control group (n = 3). In the vaccinated groups, the animals were intramuscularly immunized with 2 mL/dose of the designated bivalent FMD vaccine (Table 1). At 28-days post-vaccination (dpv), the vaccinated and unvaccinated control pigs were challenged by intradermal inoculation at two sites (100 µL per side) in the tongue with the pig-derived viruses (1 × 10^6^ TCID_50_/100 µL of A/ASIA/G-VII field isolate). All animals were challenged with the A/TUR/13/2017 strain in trial 1, while the A/BHU/3/2017 strain was used for the challenge in trial 2. After challenge, the clinical signs and lesions were monitored daily and recorded as described previously [16]. Once clinical lesions appeared in the vaccinated groups, immediate separation of the infected pig was conducted from other pigs. Sera and oral swabs were collected daily from 0- to 7-days post-inoculation (dpi). Animals that developed typical FMD manifestation at sites other than the tongue were considered unprotected. In trial 1, two animals, one from the group vaccinated with the vaccine containing A/Zabaikalsky and one from the unvaccinated control group, were excluded from the challenge study because they died before the challenge.

### 2.4. Detection of FMDV by RT–qPCR

The viral load for each individual animal was measured via real-time RT–PCR using the extracted viral RNA from the sera and oral swabs. RNA extraction was performed using the cador Pathogen 96 QIAcube HT kit in the QIAcube HT system (Qiagen, Hilden, Germany). The viral loads in sera and oral swabs were measured (in terms of copy number) using an AccuPower FMDV Real-time RT–PCR kit (Bioneer, Daejeon, Republic of Korea) according to the manufacturer’s instructions. The viral loads are expressed as log_10_ RNA copies/0.1 mL.

### 2.5. Serological Responses in Efficacy Tests

Virus neutralization (VN) titers against FMDVs in all sera were measured by VNT as described in the WOAH Terrestrial Manual 2022 [9]. Briefly, inactivated sera were diluted serially. Next, 100 TCID_50_ of the appropriate FMD vaccine or challenge viruses were added to measure the homologous or heterologous VN titers for sera from the corresponding vaccination challenge tests and incubated at 37 °C for 1 h. LFBK cells were added to the microplate and then incubated at 37 °C for 72 h with 5% CO_2_ in a humidified atmosphere to identify the cytopathic effect. The VN titer was expressed as the final log_10_ of reciprocal serum dilution for 50% neutralization of 100 TCID_50_ of the virus. Additionally, the relatedness of A/ASIA/G-VII field isolates with vaccine strains was also measured by dividing the heterologous VN titer by the homologous VN titer using porcine vaccinal sera derived from in vivo vaccination challenge experiments at 28 dpv.

### 2.6. Statistical Analysis

For the analysis of the protection status in vaccinated groups, a mixed-effects model was used to assess the efficacy of vaccination, with the vaccines, challenge viruses, and interaction between vaccines and challenge viruses as fixed effects, and individual pigs as a random effect. Overall clinical scores and RNA levels of sera and oral swabs were compared using two-way ANOVA.

For the analysis of the viral RNA levels, a mixed-effects model was used to assess the overall effects of vaccination on the viral RNA level during the experiment, with the three vaccination/protection status groups (unvaccinated control, vaccinated/not protected, and vaccinated/protected), time, and interaction between vaccination/protection status group and time as fixed effects, and individual pigs as a random effect.

To compare the total RNA levels of sera and oral swabs, the area under the curve (AUC) was calculated using the trapezoid rule. AUCs for the three groups of animals (i.e., unvaccinated, vaccinated/not protected, and vaccinated/protected) were compared using ANOVA.

In all models, Tukey’s multiple comparison test was followed to identify differences between groups for post hoc analysis.

All analyses were performed on the logarithm (base 10) of the reciprocal titers. All the statistical analyses were performed using GraphPad Prism version 10.2.0 (GraphPad, San Diego, CA, USA). *p* Values < 0.05, <0.01, <0.001, or <0.0001 were regarded as significant or highly significant.

## 3. Results

### 3.1. Vaccine Matching by 2D-VNT

According to the r_1_-values, the geometric mean of r_1_-values of the A/TUR/13/2017 strain with A/Iraq, A/Cruzeiro, and A/Zabaikalsky were 0.14, 0.41, and 0.05, respectively. The geometric mean of r_1_-values between the A/BHU/3/2017 strain and A/Iraq, A/Cruzeiro, and A/Zabaikalsky were 0.08, 0.20, and 0.03, respectively (Table 2).

### 3.2. Clinical Signs

**Figure 1:**
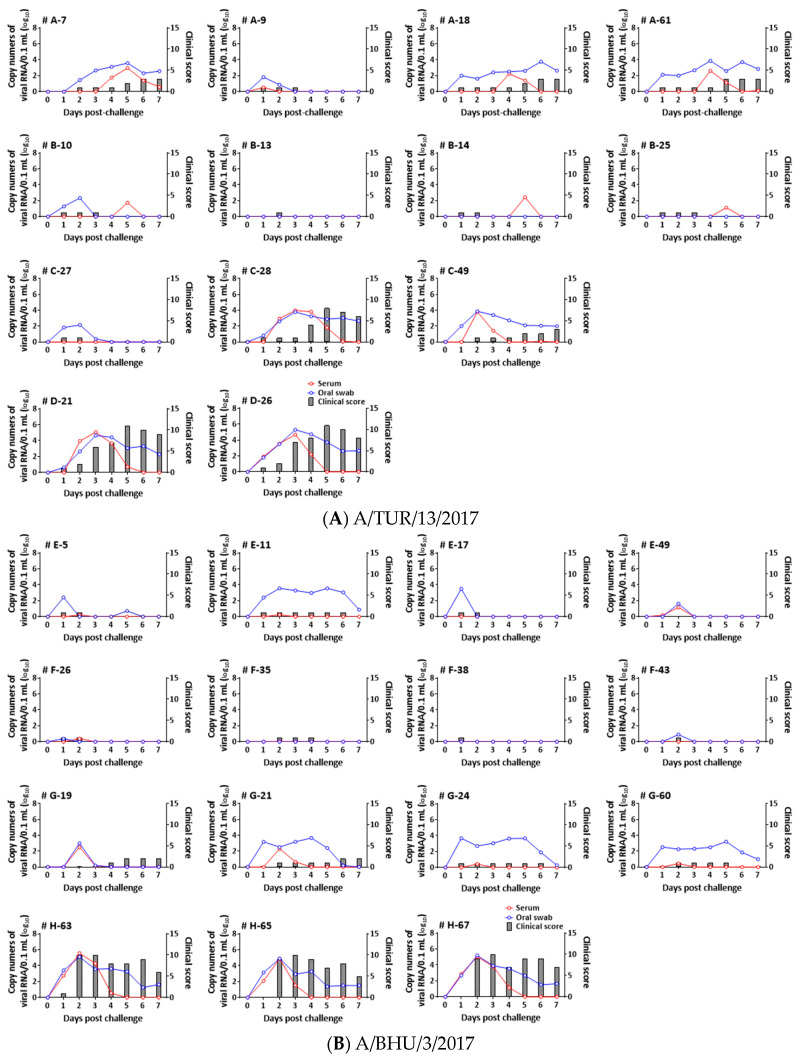
Changes in clinical scores and FMDV RNA levels in pigs immunized with the O/Manisa + O/3039 + A/Iraq, O/Campos + A/Cruzeiro+A2001, and O/Primorksy + A/Zabaikalsky vaccines after challenge with A/ASIA/G-VII lineage viruses: (**A**) A/TUR/13/2017; (**B**) A/BHU/3/2017. The number at the top left of each graph indicates the pig ID.

The clinical results of vaccination-challenge efficacy trial 1 against A/TUR/13/2017 are described in Figure 1 and Table 3. In trial 1, all unvaccinated controls (Group D) developed typical FMD lesions at sites other than the tongue within three days after challenge. Only one animal each in groups A and C was protected after challenge, while all animals in group B were protected after challenge.

The clinical results of vaccination-challenge efficacy trial 2 against A/BHU/3/2017 are described in Figure 1 and Table 4. In trial 2, none of the animals in groups E and F showed typical FMD lesions at sites other than the tongue, but two animals in group G developed clinical signs at the snout or foot. All unvaccinated controls (group H) developed clinical lesions at sites other than the tongue within 2 days after challenge.

In the vaccinated and challenged animals in both trials, the onset of clinical manifestations in all unprotected pigs was delayed, and the clinical scores in most unprotected pigs were lower than those in unvaccinated control animals (Table 3 and Table 4).

According to analysis of the mixed effect model in protection, only vaccines among the fixed effects were statistically significant [F_(3, 20)_ = 8.893, *p* = 0.0006]. The challenge virus [F_(1, 20)_ = 2.878, *p* = 0.1053] or the interaction between vaccines and challenge viruses [F_(3, 20)_ = 1.963, *p* = 0.1521] were not statistically significant. The significant difference between A/Cruzeiro + A/2001 and A/Iraq vaccinated groups (*p* = 0.0295) and between A/Cruzeiro + A/2001 vaccinated group and unvaccinated control group (*p* = 0.0165) was observed in trial 1. And the significant differences between A/Iraq vaccinated group and unvaccinated control group (*p* = 0.0061) and between A/Cruzeiro + A/2001 vaccinated group and unvaccinated control group (*p* = 0.0061) were observed in trial 2.

According to the ANOVA, significant differences in clinical scores were observed between all vaccinated groups and the control group in both efficacy trials (*p* < 0.0001). No significant differences between A/Iraq and A/Zabaikalsky vaccinated groups in trial 1 (*p* = 0.546) and between A/Iraq and A/Cruzeiro + A/2001 vaccinated groups in trial 2 (*p* = 0.898) were observed in clinical scores, although significant differences between other vaccinated groups were observed (*p* < 0.0184).

### 3.3. Detection of FMDV by qRT–PCR

In trial 1, FMD viral RNA from sera and oral swabs was detected sporadically even in protected animals, although the peak copy numbers of viral RNA were less than 10^2.5^. Viral RNA was not detected in only one animal in group B. Viral RNA was detected in the sera of unprotected vaccinated and challenged animals for at least two consecutive days, and the peak copy numbers of viral RNA in the sera and oral swabs ranged from 10^2.2^ to 10^4.0^ and from 10^3.8^ to 10^3.9^, respectively, while the peak copy numbers of viral RNA in the sera and oral swabs from unvaccinated control animals (group D) ranged from 10^4.7^ to 10^5.1^ and from 10^4.6^ to 10^5.3^, respectively (Figure 1A).

In trial 2, the peak copy numbers of viral RNA in sera from the protected animals in the vaccinated groups were less than 10^1.2^. Viral RNAs in sera from unprotected animals in the vaccinated groups were also detected for two consecutive days, but the peak copy numbers for these in the viral RNA group ranged from 10^2.3^ to 10^2.5^. The peak copy numbers of viral RNA in sera from unvaccinated control animals ranged from 10^4.9^ to 10^5.6^. Although viral RNAs in oral swabs were also detected in protected and nonprotected animals, more constant detection of viral RNA tended to be observed in the nonprotected animals after challenge. All animals in the control group (group H) tended to have greater viral loads in their sera and oral swabs than did the animals in the vaccinated groups (Figure 1B).

According to the ANOVA of viral FMD RNA levels in sera and oral swabs, significant differences were observed between all vaccinated groups and the control group in trial 1 (*p* < 0.0070) and in trial 2 (*p* < 0.0043).

### 3.4. Serological Responses in Efficacy Tests

**Figure 2:**
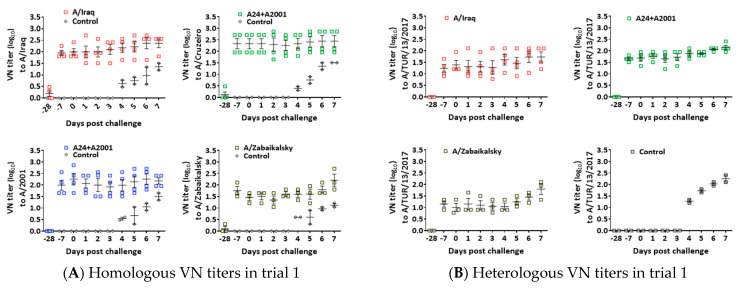

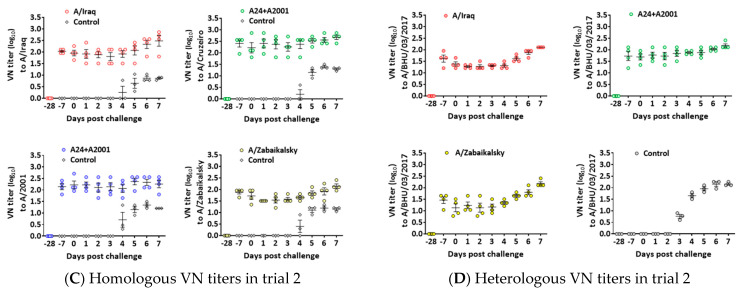
Changes in homologous and heterologous VN titers during the experiments. (**A**) The homologous VN titers were assayed in the efficacy trial after challenge with the A/TUR/13/2017 virus, and (**B**) VN titers against the challenge virus were assayed. (**C**) The homologous VN titers were assayed in the efficacy trial with A/BHU/3/2017, and (**D**) VN titers against the challenge virus were assayed. The legend at the top of each graph indicates the applied vaccine. The strain used for VNT is described to the left of Y-axis of each VNT plot. The error bars represent the SEMs.

At three weeks post-vaccination, the homologous VN titers in the vaccinated groups were maintained during the experiments (Figure 2). The average homologous VN titers in the vaccinated groups were approximately 1.95 (log_10_) or greater on the day of challenge, except those in groups C and G, which were approximately 1.5 or greater at the time of challenge. Overall, the average VN titers against the homologous antigens in trial 1 (Figure 2A) were very similar to those of the counterpart groups in trial 2 (Figure 2C). All control animals showed detectable VN titers at three- or four-days post-challenge (dpc), and the mean values reached 2.0 VN titers against the challenge strains at six dpc (Figure 2B,D).

According to the r_1_-values calculated using porcine sera collected at 28 dpv in this study, the r_1_-values of the A/TUR/13/2017 strain with A/Iraq, A/Cruzeiro, and A/Zabaikalsky were 0.25, 0.23, and 0.36, respectively. The r_1_-values between the A/BHU/3/2017 strain and A/Iraq, A/Cruzeiro, and A/Zabaikalsky were 0.27, 0.30, and 0.26, respectively (Table S1).

### 3.5. Comparison of Genome Detection in Unprotected and Protected Animals

**Figure 3:**
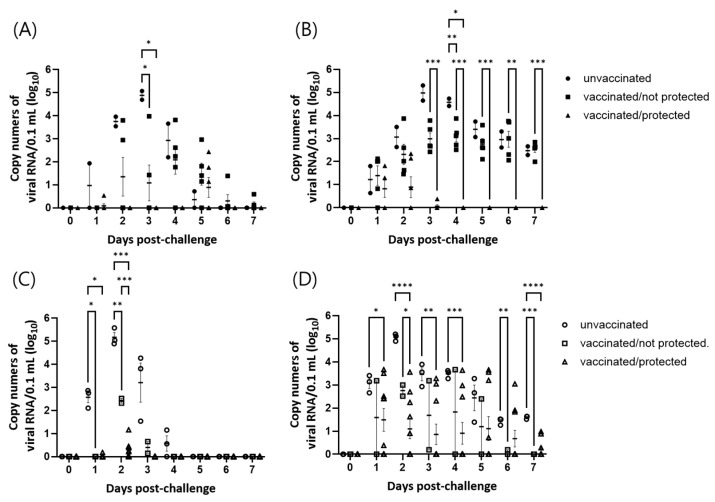
Comparison of daily viral RNA levels over time in pigs challenged with A/ASIA/G-VII lineage viruses. The daily levels of viral RNA in the sera (**A**) and the oral swabs (**B**) were depicted for the three groups of animals (unvaccinated, vaccinated/not protected, and vaccinated/protected) in trial 1 challenged with A/TUR/13/2017 strain. And the daily levels of viral RNA in the sera (**C**) and the oral swab (**D**) were depicted for the three groups of animals in trial 2 challenged with A/BHU/3/2017 strain. Error bars represent the SEMs. *, *p* < 0.05; **, *p* < 0.01; ***, *p* < 0.001; ****, *p* < 0.0001.

The daily levels of FMDV RNA in the sera and oral swabs are shown in Figure 3. According to the daily results, the levels of FMDV RNA in both samples of the unvaccinated groups reached peak levels at 3 and 2 dpc in trials 1 and 2, respectively. The levels of FMDV RNA in the vaccinated and protected groups were lower than those in the unprotected groups. In trial 1, significant differences in the levels of viral RNA between the unvaccinated control and vaccinated groups were detected at 3 dpc in the serum samples and at 4 dpc in the oral swabs. Significant differences in viral RNA levels between the vaccinated/not protected and vaccinated/protected groups were observed at 3 to 7 dpc in oral swabs.

In trial 2, significant differences in viral RNA levels between the unvaccinated and vaccinated/protected groups were detected at 1 to 2 dpc in serum samples and at 1 to 4 and 7 dpc in oral swabs. Significant differences in viral RNA levels between the unvaccinated and vaccinated/not protected groups were observed at 1 to 2 dpc in serum samples and at 6 to 7 dpc in oral swabs. Significant differences between vaccinated/not protected and vaccinated/protected groups were observed at 2 dpv in serum and oral swabs.

**Figure 4:**
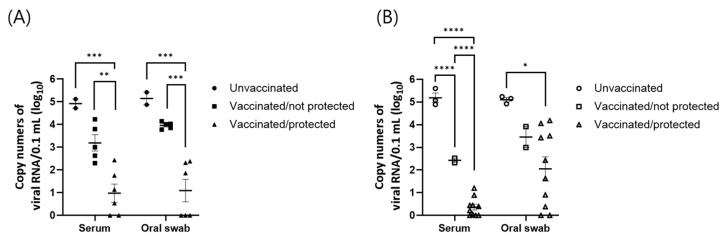
Comparison of overall viral RNA levels in pigs challenged with A/ASIA/G-VII lineage viruses. The area under curves (AUC) of both samples (**A**) were depicted for the three groups of animals (unvaccinated, vaccinated/not protected, and vaccinated/protected) in trial 1 challenged with A/TUR/13/2017 strain. And the AUC of both samples (**B**) were depicted for the three groups of animals in trial 2 challenged with A/BHU/3/2017 strain. Error bars represent the SEMs. *, *p* < 0.05; **, *p* < 0.01; ***, *p* < 0.001; ****, *p* < 0.0001.

According to the ROC analysis in trial 1, significant differences in total viral RNA loads were observed between the unvaccinated and vaccinated/protected groups and between the vaccinated/not protected and vaccinated/protected groups (Figure 4A).

In trial 2, significant differences in the total viral RNA load among all groups were observed in sera, while significant differences in total viral RNA load between unvaccinated and vaccinated/protected groups were observed in oral swabs, according to ROC analysis (Figure 4B).

## 4. Discussion

In the present study, we conducted a vaccination challenge study for estimation of cross-protection against A/ASIA/G-VII lineage viruses by administering three commercial vaccines used in Korea to pigs, which represent livestock animals that are more commonly subjected to FMD vaccination than cattle in Korea. According to the efficacy results, clinical signs were significantly alleviated in vaccinated pigs compared to the unvaccinated control groups after challenge, although there were some differences in clinical scores depending on the vaccine. A greater number of vaccinated individuals seemed to show protection, with more reduced clinical scores in vaccinated pigs, in trial 2 than in those in trial 1, while there was no clear statistical effect of challenge strains according to analysis of efficacy results using a mixed-effects model.

According to previous studies evaluating vaccines against heterologous challenge with FMD A/ASIA/G-VII lineage viruses in cattle [17,18], most commercial FMD vaccines used in the Middle East were not matched with A/ASIA/G-VII lineage viruses according to a vaccine matching test. Although the A/MAY/97 strain showed better potency than the A/Iraq vaccines in vaccination challenge tests, the serological relatedness between A/MAY/97 and the field strains A/ASIA/G-VII and A/IRN/22/2015 ranged from 0.15 to 0.7 depending on the test sera [18]. According to the PD_50_ results of the same study, the cross-protection ratio between A/MAY/97 and the field strain A/ASIA/G-VII was 0.05 or 0.04, indicating poor matching with A/IRN/22/2015. However, its PD_50_/dose against heterologous challenge was calculated to be 6.5 or 11, which is more than 3 PD_50_/dose_,_ the standard potency of the FMD vaccine in the WOAH terrestrial manual. In the present study, the r_1_-value of A/Cruzeiro strain was 0.41 with A/TUR/13/2017 and 0.20 with A/BHU/3/2017, while those of the other vaccine type A strains, A/Iraq and A/Zabaikalsky, ranged from 0.03 to 0.15 for both A/ASIA/G-VII strains in the 2D-VNT vaccine matching test using bovine vaccinal sera, as described in the WOAH terrestrial manual (Table 2). In the efficacy study, the O/Campos + A/Cruzeiro + A/2001 vaccine provided full protection against two A/ASIA/G-VII lineage viruses in vaccinated pigs, while unprotected animals were observed in groups vaccinated with O/Manisa + A/Iraq and O/Primorsky + A/Zabaikalsky vaccines. For this reason, the efficacy results seemed to match the r_1_-values of the vaccine and challenge strains. However, the r_1_-values obtained using 4-week post-vaccination sera derived from porcine heterologous challenge tests ranged from 0.23 to 0.36 and did not show any large differences between vaccine strains (Table S1). As a result, unlike the vaccine matching results obtained using bovine vaccinal sera, it could be concluded that the homologous VN titers induced by each vaccine strain had an equivalent effect on the heterologous VN titers in these porcine vaccination challenge tests. However, due to the small sample size of animals in vaccinated groups, there exists a limitation in comparing the efficacy between vaccines. With this in mind, A/Cruzeiro + A/2001 vaccines showed significant results in both trials. In addition, if heterologous VN titers against the A/ASIA/G-VII strains might be expected to increase along with highly homologous VN titers in countries or regions where repeated FMD vaccinations are conducted [19], vaccines could be used as prophylactic measures against FMD.

In the present study, FMDV RNA was also examined in sera and oral swabs. FMDV RNA was detected in some protected animals and all unprotected animals. It is well known that currently available commercial FMD vaccines do not confer sterile immunity against FMDV challenge to vaccinated animals [20,21,22]. For FMD vaccination efficacy, the WOAH terrestrial manual indicates that only clinical lesions, such as vesicles and erosion, are indicators of protection status [9]. Although viral shedding cannot be subjected to the assessment of FMD vaccine efficacy, viral assays for sera or for nasal or oral swabs are often used to obtain supplementary data that could indicate the strength of infection or possible virus shedding. According to the comparison of viral RNA levels over time (Figure 3), vaccination may reduce the levels of viral RNA over time, as significant differences between the sera and oral swabs of the unvaccinated control and vaccinated/unprotected groups were detected. However, a significant reduction in total viral RNA levels after vaccination was detected only in the serum samples of trial 2, while significant differences in total viral RNA levels were detected between the vaccinated/protected and vaccinated/not protected groups in both trials (Table 4). Therefore, it could be concluded that vaccination might reduce the viral levels in sera and oral swabs, but a clear reduction in viral RNA would be observed in vaccinated and protected animals.

Given that pigs play an important role as amplifiers of FMDV, releasing 3000-fold more aerosol virus than cattle within 24 h, especially in intensive farming areas [23], housing pigs in groups in FMD challenge studies may cause immense superinfection by amplifying aerosol virus after challenges. Therefore, challenging individuals in separate fens represents the best approach to avoiding secondary infection via aerosol virus generated by other FMD infected pigs. However, most pig farms in Korea have a very intensive population of pigs. Because commercial vaccines are used in these intensive farming situations, the protective efficacy of the FMD vaccines needs to be evaluated in housing pigs in groups, although evaluation of efficacy where pigs are challenged in separate rooms is necessary to assess the exact efficacy of the vaccine.

## 5. Conclusions

In the present study, it was demonstrated that the bivalent vaccine containing A/Cruzeiro + A/2001 may be suitable to prevent the introduction of A/ASIA/G-VII lineage viruses in pigs. Although unprotected animals were observed in groups vaccinated with two other bivalent vaccines containing A/Iraq or A/Zabaikalsky, the clinical scores and viral RNA levels in the sera and oral swabs of vaccinated animals were significantly lower than those in the sera and oral swabs of unvaccinated controls in that these two vaccines could help to control FMD outbreaks. Because higher homologous and heterologous VN titers in pigs are expected due to routine vaccination campaigns conducted by Korean governments, outbreaks of A/ASIA/G-VII isolates in the field could be appropriately controlled by vaccinating pigs with three commercial vaccines, unless herd immunity in pigs has not developed due to inappropriate vaccination.

## Figures and Tables

**Table 1:** Experimental design of three FMD vaccine efficacy studies.

Trial	Group	Vaccine Strain	No. of Animals	Challenge Strain	Day of Challenge (dpv ^1^)	Blood Collection Day (dpc ^2^)	Oral Swab Sampling Day (dpc)
1	A	O/Manisa + O/3630 + A/Iraq	4	A/TUR/13/2017	28	−28, −7, 0–7	0–7
	B	O/Campos + A/Cruzeiro + A/2001	4				
	C	O/Primorksy + A/Zaikalsky	4				
	D	Unvaccinated	3				
2	E	O/Manisa + O/3630 + A/Iraq	4	A/BHU/3/2017	28	−28, −7, 0–7	0–7
	F	O/Campos + A/Cruzeiro + A/2001	4				
	G	O/Primorksy + A/Zaikalsky	4				
	H	Unvaccinated	3				

^1^ days post-vaccination; ^2^ days post-challenge.

**Table 2:** Levels of serological relatedness between different vaccine antigens and A/ASIA/G-VII field isolates determined using bovine vaccinated sera.

Vaccine Virus	Field Virus							
	A/TUR/13/2017				A/BHU/3/2017			
	VN Titer (log_10_)		r_1_	Matching	VN Titer (log_10_)		r_1_	Matching
	HM ^1^	HT ^2^			HM	HT		
A/Iraq	2.21 2.46 2.48	1.38 1.35 1.81	0.14 ^3^	Nonmatched	2.06 2.51 2.62	1.21 1.41 1.31	0.08	Nonmatched
A/Cruzeiro	2.59 2.73 2.97	2.20 2.35 2.57	0.41	Matched	2.62 2.92 2.89	1.90 2.37 2.05	0.20	Nonmatched
A/Zabaikalsky	2.57 2.61 2.90	1.27 1.20 1.58	0.05	Nonmatched	2.66 2.96 2.71	1.15 1.18 1.11	0.03	Nonmatched

^1^ HM: Homologous VN titer; ^2^ HT: Heterologous VN titer; ^3^ Geometric mean.

**Table 3:** Day of onset of clinical manifestation after challenge with the FMDV A/TUR/13/2017 strain.

Group	Vaccine Strain	Pig No.	Lesion Development			Vesical Development				Protection
			Tongue	Lip or Gum	Snout	Forelimb		Hindlimb		
						Right	Left	Right	Left	
A	O/Manisa + O/3630 + A/Iraq	# 7	2	-	-	6	5	-	5	No
		# 9	1	-	-	-	-	-	-	Yes
		# 18	1	-	-	5	-	6	6	No
		# 61	1	-	-	-	5	6	5	No
B	O/Campos + A/Cruzeiro + A/2001	# 10	1	-	-	-	-	-	-	Yes
		# 13	2	-	-	-	-	-	-	Yes
		# 14	1	-	-	-	-	-	-	Yes
		# 25	2	-	-	-	-	-	-	Yes
C	O/Primorksy + A/Zaikalsky	# 27	1	-	-	-	-	-	-	Yes
		# 28	1	5	-	5	5	5	4	No
		# 39 ^1^	-	-	-	-	-	-	-	-
		# 49	2	-	-	6	-	7	-	No
D	Unvaccinated	# 21	1	3	3	3	3	3	3	No
		# 26	1	5	3	3	3	3	3	No
		# 43 ^1^	-	-	-	-	-	-	-	-

^1^ Excluded animals from the challenge study.

**Table 4:** Day of onset of clinical manifestation after the challenge with the FMDV A/BHU/3/2017 strain.

Group	Vaccine Strain	Pig No.	Lesion Development			Vesical Development				Protection
			Tongue	Lip or Gum	Snout	Forelimb		Hindlimb		
						Right	Left	Right	Left	
E	O/Manisa + O/3630 + A/Iraq	# 5	1	-	-	-	-	-	-	Yes
		# 11	1	-	-	-	-	-	-	Yes
		# 17	1	-	-	-	-	-	-	Yes
		# 49	-	-	-	-	-	-	-	Yes
F	O/Campos + A/Cruzeiro + A/2001	# 26	1	-	-	-	-	-	-	Yes
		# 35	2	-	-	-	-	-	-	Yes
		# 38	1	-	-	-	-	-	-	Yes
		# 43	2	-	-	-	-	-	-	Yes
G	O/Primorksy + A/Zaikalsky	# 19	1	-	5	-	-	-	-	No
		# 21	1	-	-	6	-	-	-	No
		# 24	1	-	-	-	-	-	-	Yes
		# 60	1	-	-	-	-	-	-	Yes
H	Unvaccinated	# 63	1	2	1	2	2	2	2	No
		# 65	1	2	2	2	2	2	2	No
		# 67	1	2	3	2	2	2	2	No

## Data Availability

All the data generated and/or analyzed during this study are included in this manuscript. The raw data are available from the corresponding author upon reasonable request or in the Supplementary Materials.

## Supplementary Materials

The following supporting information can be downloaded at https://www.mdpi.com/article/10.3390/vaccines12050476/s1, Table S1: Levels of serological cross-reactivity between different vaccine antigens and A/ASIA/G-VII isolates using porcine sera from vaccinated pigs collected on the challenge day.
