# Supplementary material for: Evaluation of Swine Protection with Three Commercial Foot-and-Mouth Disease Vaccines against Heterologous Challenge with Type A ASIA/G-VII Lineage Viruses
Source: Vaccines (Basel). 2024 Apr 29;12(5):476. doi: 10.3390/vaccines12050476 (PMC11125601; doi:10.3390/vaccines12050476)
Supplement: Supplementary file 1 [file vaccines-12-00476-s001.zip › vaccines-2949392-supplementary.pdf]

Table S1. Levels of serological cross-reactivity between different vaccine antigens and A/ASIA/G-VII isolates using porcine sera from vaccinated pigs collected at the challenge day.

Vaccine virus	Field virus							
	A/TUR/13/2017				A/BHU/3/2017			
	VN titer (log_{10})		r_1	Matching	VN titer (log_{10})		r_1	Matching
	HM[1]	HT[2]			HM	HT		
A/Iraq	1.95	1.20	0.25 [3]	Non-matched	1.95	1.34	0.27	Non-matched
	2.41	1.95			1.95	1.34		
	1.81	1.20			2.26	1.65		
	1.81	1.20			1.65	1.20		
A/Cruzeiro	2.71	1.95	0.23	Non-matched	1.81	1.34	0.30	Doubtful
	1.95	1.34			2.11	1.65		
	1.95	1.81			2.86	1.95		
	2.71	1.65			2.11	1.81		
A/2001	2.86	1.95	0.27	Non-matched	1.95	1.34	0.29	Non-matched
	1.65	1.34			2.11	1.65		
	2.11	1.81			2.71	1.95		
	2.41	1.65			2.11	1.81		
A/Zabaikalsky	1.65	1.34	0.36	Matched	1.34	0.78	0.26	Non-matched
	1.51	0.78			1.65	0.90		
	1.20	0.90			1.95	1.34		
	-	-			1.95	1.51		

1. HM: Homologous VN titer; 2. HT: Heterologous VN titer; 3. Geometric mean
